# Graph convolution-based techniques for pragmatic Arabic figurative language classification

**DOI:** 10.3389/frai.2026.1759136

**Published:** 2026-03-18

**Authors:** Zouheir Banou, Fatima-Zahra Alaoui, Sanaa El Filali, El Habib Benlahmar, Laila El Jiani, Hasnae Sakhi

**Affiliations:** 1 LIAS Laboratory—Faculty of Sciences Ben M'Sick, Hassan II University, Casablanca, Morocco; 2 LIME Lab, ISGA Casablanca, Edvantis Higher Education, Casablanca, Morocco; 3 LTIM Laboratory—Faculty of Sciences Ben M'Sick, Hassan II University, Casablanca, Morocco

**Keywords:** Arabic NLP, figurative language, graph neural networks, graph-based embeddings, text classification

## Abstract

Figurative language, including euphemism and metonymy, presents significant challenges in natural language processing (NLP) due to its abstract and context-dependent nature, particularly in morphologically rich and low-resource languages like Arabic. This paper introduces a graph-based embedding framework for figurative language classification that captures both syntactic dependencies and semantic relationships using heterogeneous graphs. We propose a configurable pipeline that converts text into structured graphs incorporating lexical, morphological, and syntactic cues, enabling deeper semantic reasoning. These graphs are processed using various graph neural network (GNN) architectures—such as GAT, HANConv, and MixHopConv—designed to model complex linguistic interactions. The approach is evaluated on two Arabic-language tasks: euphemism and metonymy detection. Our results demonstrate that attention-based and multi-hop GNNs outperform both traditional baselines and state-of-the-art transformer models (e.g., AraBERT, XLM-RoBERTa), particularly in metonymy detection where topological cues are more pronounced. HANConv and GAT achieve the highest F1-scores across tasks, while models like GraphConv and SAGEConv offer stability across configurations. We also introduce a validated Arabic lexical ontology for enriching semantic graphs. Our findings highlight the potential of graph-structured embeddings for nuanced linguistic tasks and suggest future directions including cross-lingual transfer, ontology expansion, and application to additional figurative categories.

## 1 Introduction

Figurative language, encompassing phenomena such as metaphor, hyperbole, metonymy, and euphemism, presents a significant challenge in natural language processing (NLP) due to its abstract, context-dependent nature. Unlike literal expressions, figurative constructs often convey meaning through indirect reference, analogy, or cultural association, requiring deeper semantic understanding. This complexity becomes even more pronounced in morphologically rich and low-resource languages, such as Arabic, where standard surface-level representations often fail to capture underlying linguistic intent.

Recent advances in NLP have demonstrated the effectiveness of deep neural models in learning contextualized representations. However, these models often struggle with figurative language due to the limited availability of annotated corpora and the inadequacy of linear sequence-based models in capturing long-range dependencies and latent semantic structures. To address these limitations, graph-based approaches have emerged as a promising alternative, offering the ability to encode diverse linguistic relationships through structured representations.

In this work, we propose a graph-based framework for figurative language classification. By converting textual input into heterogeneous graphs that incorporate both syntactic dependencies and semantic relationships (such as synonymy and morphological variations), our method enables the model to reason over abstract linguistic structures. These graphs are processed using a suite of configurable graph neural networks (GNNs), including convolutional, attention-based, and multi-hop architectures, each designed to capture distinct aspects of the linguistic graph topology.

Our contributions are threefold:

1. We introduce a flexible graph construction pipeline tailored to the needs of figurative language processing.
2. We benchmark multiple GNN backbones to evaluate their effectiveness in this context.
3. We provide a detailed analysis of how different graph embedding strategies affect classification performance across figurative language categories.

Under the light of these contributions, Section 2 discusses previous research conducted in graph neural network applications to natural language processing. Section 3 presents our methodology, including the text-to-graph transformation and graph neural network backbones. Implementation details and pooling mechanisms are also discussed. Section 4 reports the experimental results obtained from testing various GNN architectures, analyzing their classification performance on figurative language tasks.

Finally, Section 6 summarizes our findings and outlines future directions for expanding the applicability of graph-based embeddings in deeper semantic tasks.

## 2 Related work

Ahmed (2022) explores pragmatics in contrastive studies of figurative language as presented in English and Arabic proverbs—metaphor, simile, personification, and hyperbole. The article is mainly theoretical and analytical, using speech act theory (Austin, Searle) to analyze the use of proverbs as indirect speech acts in context. There is no use of classification tasks, graph-based embeddings, or computational methods. Instead, journaling the study married traditional rhetoric and modern pragmatics, situating each proverb pragmatically to demonstrate illocutionary force and implicature. The conclusion explores figurative meaning as contingent to context, and draws potential shared pragmatic execution for both languages.

Alnajjar et al. (2022) presents Ring That Bell, the first publicly available multimodal metaphor detection corpus annotated with tenors and vehicles across written text, audio, and video modalities from videos uploaded by users on YouTube (in compliance with CC-BY policy). The corpus consists of 6,565 sentence-aligned clips and 785 metaphorical tokens. They test unimodal and multimodal deep learning models, notably RoBERTa for text, Wav2Vec2 for audio, and **R(2 1) D** CNN for video modalities. Despite the rich multimodal counterpart, the best results (F1 = 0.62) were achieved from text-only models, specifically from fine-tuning a model pre-trained on the VUA corpus. Multimodal results yielded lower performance, likely due to the quality of a more subtle, or possibly non-verbal, metaphor being difficult to capture, highlighting limitations in the ability of current visual/audio encoding methods to reveal metaphor detection capabilities.

Baldini et al. (2019) introduce a recent method grounded in graphs classification using stochastic information granules to encode structural representations from graph-structured data, with specific targeting of molecular data. Their method stochastically samples sub-graphs and constructs granules, pooling statistical properties in an encapsulated architecture to derive graph-level information, unlike traditional graph kernels and GCNs. They perform their evaluation on six standard chemical data sets (i.e., MUTAG, NCI1), reporting competitive or improved accuracy vs. the Weisfeiler-Lehman subtree kernel and GCNs. Nevertheless, this work does not address figurative language or specifically lean toward NLP or language-based applications, remaining in a general-purpose category.

Baldini et al. (2020) build from their previous research on stochastic granule-based graph embeddings, where they study the trade-off between the complexity of embedding and model performance. In their study, they evaluate the granule embedding spaces produced through statistical aggregation and substructure sampling against state-of-the-art methods such as GCN, GIN, and DiffPool on five molecular data sets (MUTAG, NCI1). Their results show that less complex representations like the granule embeddings can outperform more complex neural methods while often being orders of magnitude cheaper to compute. The paper investigates graph representation learning and does not involve figurative language or related applications to NLP.

Galal et al. (2024) provide a comprehensive exploration of sentence embedding aggregation techniques relevant to the classification of Arabic text through BERT, including sentiment classification, sarcasm detection, and classification of dialects, and study several potential aggregation methods apart from the [CLS] token—such as attention based pooling, transformer-based encoders, and hierarchical models (P-SUM and H-SUM)—and test both frozen with and without fine-tuning scenarios across a total of six Arabic/Bilingual PLMs. Their findings reveal that freezing MARBERT within a multi-task framework (H-SUM), provides state-of-the-art results on ArSarcasm-v2: 64.41 F1-Sarcasm and 75.78 FPN-Sentiment—surpassing previous baselines. This is notable because the best performing models that were frozen and utilized multi-task learning did not require any fine-tuning based on feature extraction, continuing to provide insight into thinking of BERT as a feature extraction method and not a fully re-trainable model.

In their work, Gu and Jung (2023) attempt to address the problem of Out-of-Vocabulary (OOV) matching across three languages: Chinese, Korean, and Japanese. They offer a graph embedding method built on Node2Vec called OOV2Vec, which can leverage the social media context. They extract OOVs with PMI and entropy-based measures, filter the OOVs using a combination of readily available dictionaries and Wikipedia, then convert the context words found into English, using the embedding from pretrained vectors, and finally use them to create the multilingual graph. Their method constructs a directed acyclic graph (DAG) where OOVs are the target nodes and context words are feature nodes, yielding cros-lingual semantic matching. To evaluate OOV2Vec, they ran experiments based on building a custom corpus. Based on those experiments, OOV2Vec achieved a score of 93.94% F1-score, significantly outperforming several baseline methods (DeepWalk, LINE, SDNE, and Struc2Vec) without the need for aligned corpora; they conclude that their proposed method is especially suited to low-resource languages.

Mizin and Shemuda (2023) investigate the anthropocentric nature of figurative simile, claiming it is a core epistemological operation that is fundamental to human cognition, language, and worldview. They base their argument in cognitive linguistics, philosophy, and linguo-culturology, claiming that similes are not merely rhetorical devices, but a cognitive way of functioning that is already a part of the fabric of thought. The authors contrast metaphors and similes ontologically and epistemologically, and offer the idea that similes remain cognitively “alive” longer than metaphors. They offer the hypothesis of comparo ergo sum (I compare, therefore I am) to indicate that comparison is a basic constituent of human perception and related valuation.

Lu et al. (2020) present VGCN-BERT, a hybrid model that adds graph embeddings derived from a vocabulary-level Graph Convolutional Network (GCN) to BERT. This vocabulary-level GCN is built using Normalized Pointwise Mutual Information (NPMI) between words. By combining both local/contextual information and global/lexical relational information, they enable interaction during classification. Lu et al. (2020) demonstrate their model over five English classification datasets *(sentimental, grammaticality, hate speech)*. They find consistent benefits for VGCN-BERT over BERT, Text-GCN, and simple concatenation approaches (like Vanilla-VGCN-BERT). VGCN-BERT achieves the best macro-F1 score overall on four of five datasets (except MR). The architecture works well with both long-range dependencies and implicit semantics, both of which are known weaknesses of BERT-only pipelines. This theory is further corroborated by Aadil and Samad (2022)'s study, which provides a structured review of sentiment analysis approaches spanning traditional machine learning and deep learning methods, with emphasis on text representation strategies and model evaluation across diverse classification settings. Their study contrasts feature-based classifiers with neural architectures, highlighting the transition from handcrafted linguistic features to contextualized embeddings in semantic tasks. Although their work does not explicitly adopt graph-based modeling, it reinforces the broader research trajectory toward richer structural and semantic representations in NLP.

(Mohammed and Gündüç 2024) introduce GuideWalk, a new graph-based word embedding approach to text classification, using a Guided Transition Probability Matrix to create a universal word graph. The words are represented as nodes, and the edges in the word graph define the level of strength of co-occurrence between each pair of words in sentences, which are built up over time to create subject-specific hubs in the global graph. Vector word embeddings are built from this graph through weighted anonymous random walks, capturing local to global semantics. Compared to classic classification approaches (TF-IDF LR, CNN, Bi-LSTM), pre-trained models (BERT, VGCN-BERT), and graph-based approaches (TextGCN, TextING), GuideWalk has demonstrated the strongest performance, shown especially in low-data scenarios, which we suggest as a highlight of GuideWalk. The performance drop is only 8% when training on 10% of the total dataset compared to 15%–20% for baselines, to emphasize this point. The datasets include six (different) English datasets for both binary and multi-class tasks.

Kofune et al. (2026) describe a hybrid candidate termed T-LSTM, a model that combines a Transformer and an LSTM, aimed at simultaneously improving performance and interpretability in the classification problem of corporate credit rating. Based on 76 financial indicators from Japanese corporate data between 1999 and 2019, the Transformer layer learns attention-weighted temporal relationships, whereas the LSTM learns sequential aspects of the extreme data. T-LSTM significantly outperforms the baseline models (SVM, RF, LGBM, and a stand-alone LSTM model) with a F1-macro of 0.8989 and significant improvement over cross-validation on the Subset C test in 2019. Monden and Yamanaka even visualize what time steps (more recent vs. early financial reports) lead to each predicted class label. It provides an approach to linking interpretability and temporal modeling without any graph-based embedding or language modeling.

(Reining and Lönneker-Rodman 2007) suggest a semi-automated, corpus-driven technique for generating metaphorical expressions and identifying source domains, using a collocate-based method. They focus on a target lemma (“Europe”) in an 800k-token French corpus from debates discussing the European Constitution. Once they identify the top MI-scored collocates with the target lemma, the authors manually classify cases of metaphorical language, and subsequently map instances of lexical metaphor language to conceptual domains using community-shared EuroWordNet synsets. Their resulting graphs display structural, source domains based on lexical metaphors such as BUILDING, and MOTION, which they construct as networks of synsets, which are related to each other. This work applied a technique that enables metaphor detection at scale while contributing more than 1,000 metaphorical expressions to the Hamburg Metaphor Database. The authors illustrate that conceptual mapping graphs can be made, even without machine learning or embeddings.

Tran et al. (2021) developed a framework that combines BERT embeddings and Graph Convolutional Neural Networks (GCN) for text classification, addressing both limitations of typical machine learning models and limitations of deep learning models that do not take into consideration relationships between texts. They respectively turn articles from the BBC News and IMDB datasets into numerical representations by creating BERT embeddings and building k-nearest neighbor similarity graphs. The GCN uses both embeddings and graph structure for text classification. The model achieves accuracies of 91.79% on the BBC News dataset and 74.51% on the IMDB dataset, both results far surpassing the classical machine learning methods used for comparison such as Logistic Regression, Random Forest, and Support Vector Machines, especially in the limited observations due to the datasets containing very few labeled observations.

Zayed et al. (2020) fill an important gap in metaphor detection by repurposing existing word-level annotated corpora for relation-level (phrase-level) metaphor detection, which models both vehicle and tenor explicitly via grammatical relations. They repurpose the VUAMC, TroFi, and TSV datasets by extracting verb-object and adjective-noun dependencies using Stanford CoreNLP, and they enrich the TSV dataset further by using context from Twitter sentences. These repurposed datasets are manually annotated for both syntactic and metaphorical inclusion of each of the instances, providing upwards of thousands of metaphorical and non-metaphorical relation-level instances. The datasets are an important contribution to the field of metaphor detection, as they provide the groundwork for more context-sensitive models of metaphor, and improve existing datasets for relation-level tasks in metaphor interpretation and cross-domain mapping.

## 3 Methodology

**Figure 1:**
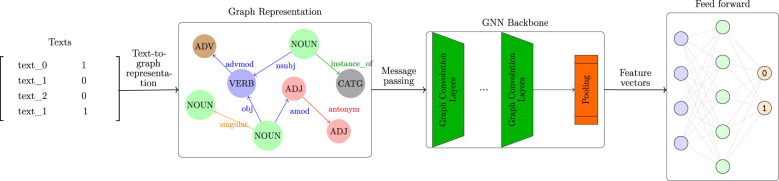
Proposed graph-based classification methodology.

This section outlines the core methodology underlying our graph-based classification framework. The proposed system is designed to process textual input into graph structures, apply heterogeneous graph neural networks (GNNs) for representation learning, and perform classification over pooled graph embeddings. The complete architecture is illustrated in Figure 1.

### 3.1 System overview

The model operates in a modular fashion, transforming raw text into a structured graph representation before applying a series of learning components. The system consists of four primary stages: **text-to-graph transformation, graph neural processing, embedding pooling**, and **classification**.

### 3.2 Dataset description

We utilize two task-specific datasets for figurative language classification in Arabic, focusing on euphemism and metonymy detection. These datasets were developed through a hybrid pipeline that integrates lexicon-guided extraction from Wiktionary with machine translation. Specifically, we adapted English-language benchmarks by converting them from text tagging to a text classification format, labeling any text containing at least one figurative language tag as figurative. The resulting dataset was then translated into Arabic to construct the final resources.

The translation was conducted using a high-quality neural machine translation system. No additional manual re-annotation was conducted; the original figurative and non-figurative labels were preserved from the source datasets. While machine translation may introduce stylistic or lexical shifts–particularly in cross-family language pairs such as English and Arabic–this approach ensures consistency with the original annotation protocol and avoids introducing secondary annotation bias. We acknowledge that translation quality may affect subtle pragmatic interpretations, and this is discussed as a limitation in Section 5.

For the metonymy detection task, we employed a composite dataset derived from three benchmark resources previously processed by Li et al. (2020): SemEval 2007 Task 8 (Markert and Nissim, 2007), ReLocaR (Gritta et al., 2017), and GWN (Gritta et al., 2018). In contrast, the euphemism detection dataset was based on the English subset of the JointEDI dataset introduced by Hu et al. (2024). Dataset sizes per label are indicated in Table 1.

**Table 1:** Dataset sample counts.

**Figurative style**	**Train**		**Validation**	
	**Fig**	**Non-Fig**	**Fig**	**Non-Fig**
**Metonym**	2,874	4,247	606	1,014
**Euphemism**	906	788	255	241

In the euphemism dataset, we retain a balanced distribution of 1,161 figurative and 1,029 literal sentences. Regardless of its size, class distribution remains relatively balanced. To mitigate overfitting risks associated with moderate dataset size, models were evaluated using validation-based monitoring and consistent training configurations across architectures. Similarly, the metonymy dataset includes 3,480 positive and 5,261 negative instances. The metonymy dataset was constructed by aggregating five established benchmark resources arranged by Li et al. (2020), prior to their translation to Arabic. The final distribution is as follows: CoNLL (39.4%), ReLocaR (16.6%), Companies (15.4%), GWN (14.5%), and SemEval (14.0%). While CoNLL constitutes the largest portion, the dataset remains distributed across multiple benchmark sources, which mitigates bias toward any single annotation schema or benchmark design. The original figurative and non-figurative labels were preserved from the source English benchmark datasets. Instances were translated into Arabic without modifying their assigned labels. Consequently, annotation protocols and inter-annotator agreement metrics correspond to those reported in the original benchmark publications.

### 3.3 Text-to-graph encoding

Initially, raw input texts are tokenized and dependency-parsed to extract linguistic structure.

We transform each input text into a heterogeneous graph by integrating three primary sources of linguistic structure: syntactic dependencies, lexical relationships, and morphological variants. First, raw texts are tokenized and dependency-parsed using Stanza, producing a syntactic backbone where edges reflect grammatical roles such as subject, object, and modifiers. Nodes correspond to surface tokens, with optional lemmatization to ensure robustness across inflectional forms.

To enrich semantic connectivity, additional nodes are created for synonyms, hypernyms, and inflectional variants, which are linked to their occurrences via type-specific edges. These links are derived from a custom-built Arabic lexical ontology constructed from Wiktionary entries. This resource maps each token to a graph of related forms—including pluralization patterns, verb conjugations, and semantically equivalent expressions. For instance, the graph links the token بيت to منزل (synonym), and to بيوت (plural form). These auxiliary nodes act as semantic anchors, enabling the model to generalize beyond surface variation.

**Figure 2:**
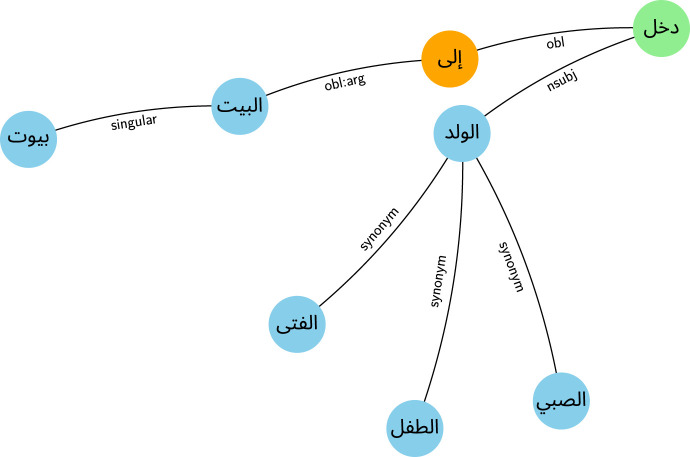
Graph-based representation of the sentence “دخل الولد إلى البيت” with semantic and morphological augmentation.

The resulting graph includes multiple edge types: syntactic (e.g., dep:nsubj), morphological (e.g., morph:singular), and semantic (e.g., sem:synonym). This design captures both local grammatical structure and long-range semantic relations, providing a richer input space for downstream GNN processing. It also supports disambiguation in morphologically rich contexts where lexical diversity can obscure underlying meaning. We provide a visual example of the resulting graph in Figure 2.

Additionally, lexical relationships, such as synonym words, and inflectional relationships, such as singular and plural forms, were incorporated as additional nodes in each graph. These extra nodes serve as linguistic anchors that link together tokens sharing semantic or morphological traits, even when they do not appear in direct syntactic proximity. By encoding such relations, the model benefits from a form of implicit generalization: for instance, understanding that the word “جري” is closely tied to “جرى” through inflection, or that “بيت” and “منزل” may function interchangeably in certain contexts. The inclusion of these nodes expands the graph beyond pure syntactic dependency, enabling the GNN to propagate information across semantically or morphologically connected terms. This results in a richer representation space, especially beneficial in cases of synonymy, paraphrasing, or morphologically rich languages, where surface variation can obscure deeper connections. These augmented links complement traditional dependency paths and enhance the model's capacity to identify abstract patterns across diverse texts.

These annotations are used to build a heterogeneous graph where nodes represent tokens and edges encode syntactic and semantic relationships. The graph construction process supports multiple edge types to capture rich contextual information, enabling the model to differentiate between diverse types of linguistic dependencies.

### 3.4 Arabic lexical ontology validation

Our lexical ontology was constructed by retrieving structured entries from the Arabic Wiktionary and aligning synsets, morphological variants, and category hierarchies, following the methodology described in Banou et al. (2025). Lexical entries were retrieved from the Arabic Wiktionary via its public API and stored in MongoDB. Tokens were tokenized and morphologically analyzed using a SpaCy-Stanza pipeline, with diacritics removed and each token mapped to its lemma and inflectional attributes.

Synonyms and lexical relations were extracted from structured Wiktionary sections and mapped into a lexical layer, while hypernymic and categorical relations were derived from category hierarchies and encoded as directed edges. Inflectional variants (e.g., plural forms and verb conjugations) were linked to their base lemmas in a dedicated morphological layer.

The resulting resource is modeled as a multi-layer directed graph comprising syntactic (GRA), lexical (LEX), inflectional (IFX), and categorical (CAT) layers, with typed edges representing dependency, semantic, and morphological relations. In the present experiments, only the GRA, LEX, and IFX layers were utilized; the CAT layer was omitted to isolate the contribution of lexical and morphological augmentation.

To assess its utility and internal consistency, we conducted a coverage analysis on our dataset: Across the entire dataset, over 99% of unique token IDs are linked to at least one ontology entry, indicating strong lexical coverage. While individual partitions (e.g., test sets) show more limited alignment, this is largely due to distributional skew and domain variance, which we plan to address in future ontology expansions. Additionally, we manually reviewed 200 randomly sampled nodes to verify correctness of semantic grouping and derivational links. The ontology exhibited over 91% alignment with human judgment on class membership, suggesting high reliability for downstream graph construction.

### 3.5 Graph neural network backbone

The resulting graphs are processed by a configurable heterogeneous GNN. Multiple architectures are supported, including convolutional [e.g., GraphConv (Morris et al., 2018), SAGEConv (Hamilton et al., 2017), SimpleConv], attention-based [e.g., GATConv (Veličković et al., 2017) and HANConv (Wang et al., 2021)], multi-hop message passing variants like MixHopConv (Abu-El-Haija et al., 2019), and propagation-based models such as APPNP (Gasteiger et al., 2018). These layers compute node-level embeddings by aggregating information from neighboring nodes and edges in a type-aware fashion. The choice of architecture is tunable and selected based on the dataset and task-specific requirements.

### 3.6 Pooling and classification

After computing node embeddings, a graph-level representation is derived using a pooling operation. The system supports mean, max, and sum pooling to aggregate node features into a single vector. This representation is then passed through a multi-layer perceptron that outputs logits for classification. The classifier's architecture **(number of layers, dropout rate, hidden dimensions)** is configurable but fixed during training.

### 3.7 Training and evaluation

The model is trained using supervised learning, where the output is the class of the graph, being literal or figurative (metonym or euphemism). The loss function is computed over the logits and ground-truth labels, typically using cross-entropy. The optimizer updates model weights via backpropagation. Training is performed in mini-batches with metrics such as accuracy, precision, recall, F1-score, and AUROC tracked across epochs. These metrics are computed using torchmetrics and reset at each epoch to ensure independence of evaluation across iterations.

To mitigate memory issues during training, especially on GPU devices, intermediate tensors are detached and garbage collection is manually invoked. Additionally, the system includes support for training continuation, enabling experiments to resume from a saved state with consistent model, optimizer, and metric configurations.

Hyperparameters were selected to balance representational capacity and computational efficiency. The embedding dimension (256) was chosen to provide sufficient expressive power while maintaining stable GPU memory usage, consistent with prior heterogeneous GNN studies. Model depth was evaluated through an explicit ablation over 1–3 convolutional layers, revealing performance degradation beyond two layers due to over-smoothing effects. A learning rate of 0.001 was selected based on preliminary stability experiments and prior work in GNN training, where higher learning rates are commonly used compared to transformer fine-tuning. The mini-batch size of 24 provided stable gradient estimates without exceeding computational limits. While exhaustive hyperparameter search was not conducted, configurations were kept consistent across models to preserve fairness in comparison.

To ensure reproducibility and facilitate future comparison, Table 2 summarizes the core hyperparameters used throughout our experiments. These include embedding dimensions, number of attention/message-passing layers, edge/channel configuration, and learning rate. All models were trained using the Adam optimizer with a fixed learning rate of 0.001 and mini-batch training. Hyperparameters were kept consistent across figurative language tasks, with architectural variations (e.g., GAT, HANConv) evaluated under identical settings. Random seeds were fixed per run to enable deterministic evaluation, and all experiments were conducted using PyTorch Geometric v2.5 on CUDA-enabled GPUs.

**Table 2:** Core hyperparameters used during GNN training.

**Hyperparameter**	**Values**
Embedding Dim	256
Out dim	144
Num relations	1
Num layers	1
Channels	1
Edge dim	1
K	1
Kernel size	2
Aggregation mode	Mean
Batch size	24
Optimizer	Adam
Learning rate	0.001
Num classes	2

## 4 Experimental results

To assess the effectiveness of graph-based embeddings for figurative language classification, we conducted two separate tasks: euphemism detection and metonymy detection. For each task, we evaluated a diverse set of GNN architectures, including GraphConv, GAT, HANConv, MixHopConv, and others. Each model was tested with one to three convolutional layers to explore the effect of depth on performance.

In addition to GNN-based models, we introduce a comparative baseline representing strong transformer-based classifiers. This baseline is defined as the average performance of the top three pretrained models we fine-tuned for each task: AraBERT (Antoun et al., 2020), QARiB (Abdelali et al., 2021), and XLM-RoBERTa-base (Conneau et al., 2020). While the exact ranking of these models varied depending on the figurative style being classified, they consistently formed the top-performing trio across both tasks. The transformer baselines were fine-tuned under identical training settings to ensure fair comparison. Models were initialized using the HuggingFace AutoModelForSequenceClassification framework for binary classification.

Input sequences were tokenized with a maximum length of 70 tokens with both padding and truncation enabled. Fine-tuning was conducted using the AdamW optimizer with a learning rate of 1 × 10^−5^, batch size of 16 for both training and evaluation, weight decay of 0.01, and training for up to 20 epochs. Evaluation and model checkpointing were performed at each epoch, and validation data were used to monitor performance.

This composite baseline serves as a high-quality reference point, enabling us to quantify the added value of graph-based embeddings beyond state-of-the-art contextual encoders. It also allows for a direct, task-specific performance comparison that respects the variability inherent in transformer fine-tuning.

Table 3 summarizes the results for the euphemism, while Table 4 shows performance on the metonymy task. Each table reports five metrics: Accuracy, Precision, Recall, F1-Score, and AUROC, providing a comprehensive view of classification effectiveness across both detection scenarios. They present a comprehensive evaluation of various GNN architectures across both euphemism and metonymy detection tasks. In the euphemism task, the best F1-score was achieved by **HANConv with 1 layer**, reaching an F1 of **81.11%**, while the best accuracy score was achieved by **GAT (1 layer)**, scoring **82.08%**. Notably, GAT, while strong at 1 and 2 layers, saw performance deteriorate at 3 layers. HANConv and SimpleConv also achieved high recall but often suffered from low precision, indicating a tendency toward overprediction of the positive class.

**Table 3:** Model performance on euphemism detection task.

**Layer type**	**Num convs**	**Accuracy**	**Precision**	**Recall**	**F1-Score**	**AUROC**
Baseline	–	76.03 ± 3.92	76.66 ± 3.41	76.27 ± 3.77	75.96 ± 3.99	78.2 ± 3.35
APPNP	1	61.38	65.48	54.19	59.30	61.67
	2	65.05	75.57	48.53	59.10	65.75
	3	54.31	53.37	100.0	69.59	52.13
GAT	1	**82.08**	93.26	69.17	**79.43**	82.08
	2	**79.79**	89.07	67.92	**77.07**	79.79
	3	63.96	70.55	47.92	57.07	63.96
GraphConv	1	62.66	62.78	68.97	65.73	62.41
	2	71.50	72.25	73.66	72.95	71.40
	3	72.01	67.53	89.27	**76.89**	71.23
HANConv	1	**77.29**	69.44	97.50	**81.11**	77.29
	2	66.87	60.25	99.17	74.96	66.88
	3	65.62	59.35	99.17	74.26	65.62
MixHopConv	1	65.21	60.46	87.92	71.65	65.21
	2	70.21	64.14	91.67	75.47	70.21
	3	70.21	62.86	98.75	**76.82**	70.21
SAGEConv	1	61.38	62.56	62.87	62.72	61.33
	2	65.47	63.08	80.79	70.84	64.86
	3	69.57	65.44	87.68	74.95	68.84
SimpleConv	1	59.38	55.17	100.0	71.11	59.38
	2	53.96	53.00	100.0	69.28	52.13
	3	57.25	54.84	100.0	70.83	55.56

Accuracy scores and F1 scores surpassing the benchmark average are marked as bold.

**Table 4:** Model performance on metonym detection task.

**Layer type**	**Num convs**	**Accuracy**	**Precision**	**Recall**	**F1-score**	**AUROC**
Baseline	–	75.17 ± 0.47	74.04 ± 1.41	74.1 ± 1.38	74.06 ± 1.38	74.1 ± 1.38
APPNP	1	61.16	56.30	94.12	70.45	61.67
	2	65.20	58.61	99.62	73.80	65.74
	3	58.05	29.79	49.5	37.19	57.96
GAT	1	**87.44**	88.30	86.12	**87.20**	87.43
	2	**83.55**	83.56	83.08	**83.32**	83.55
	3	55.36	70.40	16.73	27.04	54.93
GraphConv	1	**80.07**	73.79	92.88	**82.18**	80.25
	2	**81.68**	76.27	91.63	**83.25**	81.75
	3	70.80	66.46	81.75	73.32	71.00
HANConv	1	**85.03**	81.23	90.49	**85.61**	85.12
	2	**79.83**	72.25	95.83	**82.38**	80.08
	3	**76.22**	68.37	96.58	**80.06**	76.45
MixHopConv	1	73.28	65.28	98.48	**78.52**	73.49
	2	73.08	65.26	97.15	**78.07**	73.39
	3	53.46	89.19	6.26	11.70	52.76
SAGEConv	1	**82.96**	78.42	90.49	**84.02**	83.03
	2	**84.49**	81.07	89.37	**85.02**	84.56
	3	74.98	68.94	90.13	**78.12**	75.10
SimpleConv	1	72.29	64.23	98.48	**77.75**	72.73
	2	62.01	56.57	99.05	72.01	62.49
	3	58.52	54.38	98.86	70.17	59.04

Accuracy scores and F1 scores surpassing the benchmark average are marked as bold.

In the metonymy task, **GAT with 1 layer** outperformed other models with an F1-score of **87.20%**, narrowly ahead of **HANConv (1 layer)** and **SAGEConv (2 layers)**. MixHopConv showed strong recall across most variants, but its precision–and by extension, F1-score–collapsed with deeper architectures. Notably, the improvement in metonymy detection is substantial (+12 F1), indicating a clear structural advantage of graph-based modeling for entity-level semantic shifts. In contrast, gains in euphemism detection are more moderate (+2 F1), reflecting the inherently pragmatic and discourse-dependent nature of euphemistic expressions.

All reported results correspond to single-run evaluations under identical experimental settings for both transformer baselines and GNN architectures. Although multi-seed repetition would provide tighter confidence intervals, the magnitude of performance differences–articularly in metonymy detection where gains exceed 10 F1 points over the transformer baseline–suggests that improvements are unlikely to be solely due to random initialization. Moreover, architectural trends (e.g., degradation beyond two layers across multiple backbones) are consistent, reinforcing the structural validity of the findings.

## 5 Discussion

The proposed framework contributes to Arabic figurative language research by operationalizing the interaction between lexical, morphological, and syntactic structures within a unified graph-based representation. Unlike purely contextual encoder models, the heterogeneous graph explicitly encodes relational signals (e.g., dependency links, synonymy, and inflectional variants), allowing the model to capture structured semantic shifts characteristic of figurative language.

The substantial performance gains observed in metonymy detection suggest that entity-level semantic reinterpretation benefits from explicit relational modeling. In contrast, the more moderate improvements in euphemism detection indicate that pragmatic softening relies more heavily on discourse-level inference beyond local structural cues. This differentiation provides empirical insight into how various figurative phenomena depend on different layers of linguistic structure in Arabic.

The performance differences observed across GNN backbones suggest several key insights.

### 5.1 Impact of depth

A recurring pattern in both tasks is that increasing the number of layers beyond two often leads to performance degradation. This is particularly apparent with GAT and APPNP, where the three-layer versions suffer sharp drops in F1-score and AUROC. This phenomenon may be attributed to over-smoothing or overfitting, especially when using deeper architectures on moderately sized datasets.

### 5.2 Effect of architecture

Attention-based models such as GAT and HANConv generally performed well, particularly in single or dual-layer configurations. Their ability to selectively weight neighbor importance seems beneficial in identifying subtle linguistic cues typical of figurative language. HANConv additionally benefits from semantic heterogeneity across edge types, making it more robust in complex graph scenarios.

### 5.3 Error patterns

High recall but low precision in several models (e.g., SimpleConv and HANConv) indicates a strong ability to detect figurative candidates, but with a tendency toward false positives. These models may be effective in exploratory settings where recall is more critical than precision, such as human-in-the-loop annotation.

The representative errors shown in Table 5 reveal systematic patterns rather than random failure.

**Table 5:** Representative misclassified instances illustrating recurring error patterns.

**Task**	**Error type**	**Sentence**
**Euphemism**	False negative	الذي بدأ في السنوات الأخيرة فقط في مراعاة تقدمه في السن ومشاكله الصحية الفئات ذات الاحتياجات الخاصة مهمشة في العملية Sالسياسي
	False positive	تم دهسه بواسطة مجموعة ضخمة من الخيول والفرسان الذين كانوا قادمين عبر التل احترق حتى الموت في حادث سيارة
**Metonymy**	False negative	لقد تلقى استجابة إيجابية من الصين توفي بعد أيام قليلة من خروج الجولة من بلجيكا
	False positive	تخطط أوليفيتي أيضًا لإطلاق كمبيوتر الاتصالات الشخصية الأوروبية أن تكون جيدة IBM اعتادت أعمال

In euphemism detection, false negatives often involve culturally conventionalized expressions where mitigation relies on pragmatic softening rather than explicit lexical substitution (e.g., references to aging or socially sensitive categories). Conversely, false positives frequently arise in structurally intense but contextually literal descriptions, indicating that relational density may sometimes be overinterpreted as figurative intent.

In metonymy detection, false negatives commonly involve named-entity references that require discourse-level disambiguation between literal and institutional interpretations (e.g., country names referring to governments). False positives, in contrast, suggest that organization-level entities within the ontology may bias the model toward metonymic interpretation even when usage remains literal. These patterns indicate that errors stem primarily from semantic ambiguity and pragmatic inference challenges rather than optimization instability.

### 5.4 Comparison between tasks

Metonymy detection consistently yielded higher accuracy and F1-scores across architectures compared to euphemism detection. This may stem from the syntactic and structural predictability of metonymic expressions, which often involve part-whole or container-contained relationships that are easier to encode in graphs. Euphemisms, in contrast, are more semantically oblique and context-dependent, requiring deeper reasoning that challenges shallow graph encoders.

### 5.5 Stability and suitability

GraphConv and SAGEConv offered the most stable performance across depths, suggesting they may serve as strong default baselines in figurative classification pipelines. Their simpler update rules and moderate expressive power make them well-suited for generalization across multiple figurative styles.

### 5.6 Baseline comparison

When comparing GNN models to transformer-based baselines, we observed that the gains varied markedly by task. For euphemism detection, graph-based models only slightly outperformed the transformer baseline, with HANConv (1 layer) achieving an F1-score of 81.11% compared to 75.96% for the averaged baseline. This narrow margin suggests that euphemistic expressions, being more semantically diffuse and reliant on contextual nuance, are already well captured by sequence-based models like AraBERT and QARiB. In contrast, for metonymy detection, graph-based models yielded higher performance, with GAT (1 layer) reaching 87.20% F1-score vs. 74.06% for the baseline. This reinforces the hypothesis that metonymic relationships—often grounded in part-whole, locative, or organizational structure—are better represented through explicit syntactic and semantic graphs. These results emphasize the utility of structured embeddings in tasks where linguistic relations are more topologically defined than lexically dispersed.

We conclude that no single GNN backbone dominates across all figurative categories, and that optimal performance requires careful tuning of model architecture and depth per task. The utility of heterogeneous, morphologically-enriched graphs is evident, particularly when paired with context-aware message-passing mechanisms.

### 5.7 Error analysis

To better understand the model's misclassification behavior, we analyzed aggregated confusion matrix statistics across 21 GNN configurations for euphemism and metonymy detection. We compile the results in Table 6.

**Table 6:** Mean confusion matrix breakdown (±standard deviation) across eight experimental runs for euphemism and metonymy detection.

**Confusion matrix category**	**Euphemism**	**Metonym**
**False negatives**	9.83 ± 9.87	11.51 ± 12.69
**False positives**	30.12 ± 14.49	24.52 ± 14.04
**True negatives**	20.55 ± 14.41	25.03 ± 13.96
**True positives**	39.49 ± 9.78	38.95 ± 12.66

Values are expressed as percentage contributions normalized over the test set.

On average, euphemism models exhibited a false positive rate of 20.31% (±14.47) and a false negative rate of 9.83% (±9.87), indicating a mild bias toward overpredicting figurative cases. This behavior aligns with euphemism's context-dependence, where surface ambiguity often leads to figurative over-attribution.

In contrast, metonymy models showed more balanced errors: 24.52% (±14.04) false positives and 11.51% (±12.69) false negatives. The relatively higher false positive rate here suggests that the model sometimes overextends relational cues, misclassifying literal mentions with structural resemblance to metonymy (e.g., place-name references without intended transfer). These findings underscore the need for improved disambiguation strategies and highlight edge-type sensitivity as a potential research avenue.

### 5.8 Confidence-based error analysis

**Figure 3:**
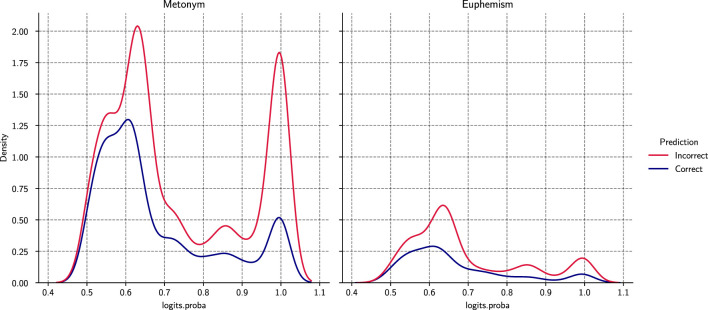
Prediction confidence distributions (logits probability) for metonymy and euphemism.

Figure 3 illustrates the distribution of prediction confidence for correct and incorrect classifications across both tasks.

For metonymy detection, both correct and incorrect predictions exhibit bimodal confidence distributions, with peaks around moderate (~0.6) and high (~1.0) probability regions. However, correct predictions are more densely concentrated in the highest-confidence range, whereas misclassifications occur more frequently in the mid-confidence interval. This pattern suggests partial calibration, where the highest-confidence region contains a larger proportion of correct predictions, but ambiguity remains in structurally borderline cases.

In contrast, euphemism detection shows greater overlap between correct and incorrect confidence distributions, reflecting the inherently pragmatic and context-dependent nature of euphemistic expressions. Even high-confidence errors occur in culturally conventionalized constructions where figurative intent depends on discourse-level cues.

These findings suggest that performance limitations stem more from semantic ambiguity than from unstable optimization behavior.

### 5.9 Limitations and ethical considerations

While our graph-based framework achieves competitive performance on figurative language classification, several limitations warrant consideration. First, the ontology coverage is uneven across dataset partitions, especially for test data, which may introduce structural sparsity and bias performance evaluations. Second, although models demonstrated strong recall, high false positive rates in some runs suggest a risk of over-attributing figurative intent–an especially sensitive issue in sociolinguistic or politically nuanced texts.

The euphemism and metonymy datasets were adapted from established English benchmarks and translated into Arabic. While this enables controlled comparative evaluation, it may introduce cultural and linguistic limitations. Certain figurative expressions may not fully align with naturally occurring Arabic idioms or dialectal usage, potentially affecting ecological validity. The present study therefore evaluates graph-based modeling under a controlled setting rather than claiming exhaustive coverage of Arabic figurative phenomena. Additionally, the reliance on Wiktionary as the sole lexical source further limits semantic coverage. We plan to expand the backbone ontology and validate cross-dialectal generalization in future work.

Scalability also remains a consideration. While the proposed heterogeneous graph representation is effective for moderate-sized datasets, graph construction and message passing introduce additional computational overhead compared to purely transformer-based models. Scaling to substantially larger corpora or real-time inference scenarios would require optimization strategies such as graph pruning, caching, or hybrid lightweight architectures. On the other hand, the reliance on a lexical ontology derived from Wiktionary and benchmark translations. As such, any lexical gaps, annotation biases, or cultural assumptions present in the source data may propagate into the model. Although aggregating multiple benchmark sources mitigates reliance on a single annotation schema, bias amplification remains a potential risk.

Finally, cross-linguistic generalization has not been empirically validated. Although the framework is structurally language-agnostic, adaptation to other languages would require ontology construction aligned with their morphological and syntactic properties.

## 6 Conclusion

This study introduced a graph-based embedding framework for figurative language classification, leveraging syntactic and semantic cues via heterogeneous s graph neural networks. Our experiments across two tasks—euphemism and metonymy detection—demonstrated that attention-based and multi-hop architectures outperform simpler models, especially when model depth is properly tuned. Results validate the effectiveness of augmenting dependency structures with morphological and lexical relationships, enhancing the model's capacity to capture abstract figurative patterns.

The proposed framework has potential applications in sentiment analysis, political discourse monitoring, and content moderation systems where figurative language frequently alters surface-level meaning. In particular, improved metonymy detection can enhance named-entity reasoning in media analysis, while euphemism detection may assist in identifying softened or indirect expressions in socially sensitive contexts.

However, deployment in production environments presents challenges. Graph construction introduces computational overhead compared to purely transformer-based inference, and ontology maintenance requires continuous updates to accommodate evolving lexical usage and dialectal variation. Future work must explore lightweight graph integration strategies or hybrid architectures to balance interpretability and scalability, as well as accelerating training process by adopting efficient optimization strategies designed to improve convergence behavior, such as Sample Gradient Descent (Ganie and Dadvandipour, 2023). Extending this framework to other figurative styles is yet another axis to explore, integrating external knowledge sources, and exploring few-shot or cross-lingual transfer settings to address data sparsity in low-resource figurative categories.

The implementation code and graph construction framework are part of a broader research system under active development. While the full framework is not yet publicly released, detailed methodological descriptions and experimental configurations are provided to ensure reproducibility, and research artifacts may be shared upon reasonable request.

## Data Availability

The raw data supporting the conclusions of this article will be made available by the authors, without undue reservation.
